# Hypermetabolic Pulmonary and Mediastinal Lesions With Elevated Cancer Antigen (CA) 15-3 and CA 27-29 in a Patient With a History of Ovarian and Breast Cancer

**DOI:** 10.7759/cureus.55712

**Published:** 2024-03-07

**Authors:** Sindhaghatta Venkatram, Maria Duran, Ked Fortuzi, Aam Baqui, Thanh-ha Luong, Gilda Diaz-Fuentes

**Affiliations:** 1 Pulmonary and Critical Care Medicine, BronxCare Health System, Bronx, USA; 2 Medicine, BronxCare Health System, Bronx, USA; 3 Pulmonary and Critical Care Medicine, Bronxcare Health System, Bronx, USA; 4 Pathology, BronxCare Health System, Bronx, USA; 5 Oncology/Hematology, BronxCare Health System, Bronx, USA

**Keywords:** cancer markers, lung lesions, ovarian cancer, breast cancer, sarcoidosis

## Abstract

Breast cancer affects around 13% of women. Breast cancer gene 1 (*BRCA1*) carriers are prone to lung and lymph node metastasis, while breast cancer gene 2 (*BRCA2*) carriers tend to have bone metastasis. Findings of pulmonary nodules, mediastinal lymphadenopathy, and elevated markers such as cancer antigen (CA) 15-3 and CA 27-29 suggest metastatic disease. Here, we present the case of a patient with *BRCA1*-positive breast cancer in remission and a history of ovarian cancer with mediastinal lymphadenopathy and pulmonary nodules, with avid fluorodeoxyglucose uptake on positron emission tomography (PET) scan and elevated CA 15-3 and CA 27-29.

A 70-year-old female with a history of bilateral breast and ovarian cancer and a positive *BRCA *test presented with pulmonary nodules, mediastinal lymphadenopathy, and elevated CA 15-3 and CA 27-29. Imaging showed mediastinal and hilar lymphadenopathy. A PET scan revealed increased metabolic activity in the lymph nodes and pulmonary lesions. Fiberoptic bronchoscopy and endobronchial ultrasound lymph node sampling demonstrated granulomatous inflammation without malignant cells. The patient underwent a therapeutic trial of steroids with clinical improvement of symptoms and decreased hypermetabolic activity in chest lesions, as well as a decrease in tumor markers.

The coexistence of sarcoidosis and breast cancer is rare; sarcoidosis can coexist, precede, or appear after breast cancer. In both conditions, tumor markers and PET avidity are seen, which makes diagnosis and management challenging. In case of ambiguity, biopsy is crucial. This case underscores the importance of integrating clinical, pathological, and imaging data to reach an accurate diagnosis and consider a therapeutic trial of steroids. Furthermore, the early PET response to treatment can be pivotal in differentiating between sarcoidosis and malignancy, especially in complex clinical scenarios. Proper differentiation is paramount to avoid therapeutic missteps and ensure appropriate patient management.

## Introduction

Breast cancer is a leading concern of women worldwide due to its high morbidity and mortality [1]. It is a complex heterogeneous group of diseases with distinct histopathological and biological subtypes that lead to differences in response to various available treatments [2]. About 13% of women in the general population will develop breast cancer sometime during their lives [3]. By contrast, 55%-72% of women who inherit a harmful breast cancer gene 1 (*BRCA1*) variant and 45%-69% of women who inherit a harmful breast cancer gene 2 (*BRCA2*) variant will develop breast cancer by 70-80 years of age [3-5]. Lung and distant lymph node metastasis is frequently seen in *BRCA1* carriers whereas *BRCA2* carriers frequently have bone metastasis [5]. In patients with a history of breast and ovarian cancer, pulmonary nodules and mediastinal lymphadenopathy usually trigger a workup for metastasis. The presence of elevated cancer antigen (CA) 15-3 and CA 27-29 and high fluorodeoxyglucose (FDG) on positron emission tomography (PET) scan points to a diagnosis of metastatic disease.

We present the diagnostic challenge in a patient with a history of breast and ovarian cancer in remission presenting with pulmonary lesions, mediastinal lymphadenopathy with avid FDG uptake, and elevated CA 15-3 and CA 27-29.

## Case presentation

**Figure 1:**
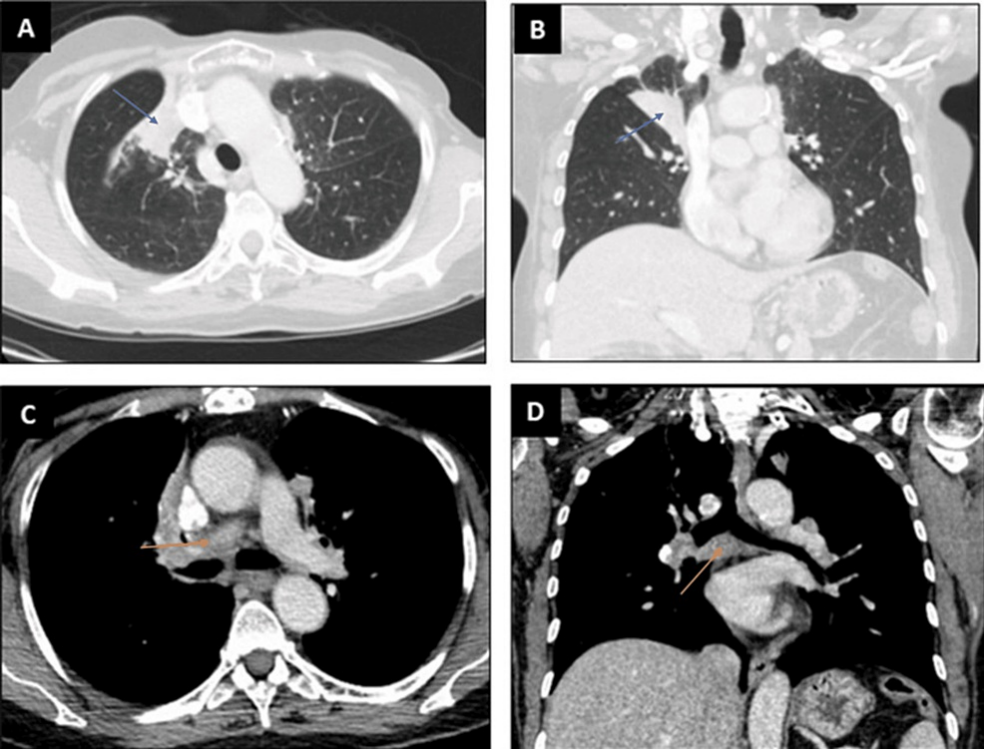
CT of the chest showing lung lesions and lymphadenopathy. (A, B) Axial and coronal view on CT scans (parenchymal window) showing right upper lobe lesion. (C, D) Axial and coronal view on CT scans showing mediastinal and hilar lymphadenopathy. (A, B) Blue arrow showing a lesion in the right upper lobe. (C, D) Orange arrow showing mediastinal lymphadenopathy.

A 70-year-old female patient from Kosovo, southeast Europe, presented to our pulmonary clinic after findings of mediastinal and hilar lymphadenopathy on CT and elevated tumor markers CA 15-3 and CA 27-29 were noted. She had a medical history of bilateral breast cancer and ovarian cancer, pulmonary tuberculosis, myocardial infarction with stent placement, and COVID-19 infection. Left breast cancer was diagnosed when she was 33 years old and living in Kosovo and was treated with left mastectomy and adjuvant radiotherapy. Left ovarian cancer was diagnosed at age 49 and treated with chemotherapy, total abdominal hysterectomy, and salpingo-oophorectomy. The patient moved to the United States, and *BRCA* testing in 2007 was positive. She declined a right mastectomy. She underwent normal screening mammograms until 2020 when a suspicious mass was found. A biopsy of the lesion confirmed invasive ductal breast cancer. She underwent a right mastectomy followed by chemotherapy. She was followed up by oncology, and during a surveillance chest CT, she was found to have wedge-shaped opacification of the lung and mediastinal lymphadenopathy (Figure 1).

A PET scan revealed multiple hilar or mediastinal lymph nodes with elevated metabolic activity (standardized uptake value (SUV): 8.2 to 14.4). Elevated metabolic activity was also noted within pulmonary lesions (SUV: 9-11.3).

**Figure 2:**
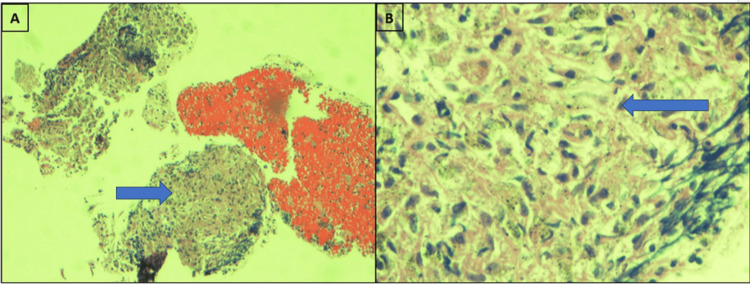
Section of lymph node at lower (10×, A) and (40×, B) magnification showing necrotizing and non-necrotizing granulomatous inflammation with scattered epithelioid histiocytes. Blue arrows showing necrotizing and non-necrotizing granulomas.

The patient underwent fiberoptic bronchoscopy with transbronchial lung biopsy (TBBX) and endobronchial ultrasound with transbronchial lymph node aspiration (EBUS-TBNA). Pathology is shown in Figure 2. The pathological diagnosis was necrotizing granulomatous inflammation, without malignant cells, and no acid-fast bacilli or fungi. All cultures were negative. The patient was offered mediastinoscopy and surgical biopsy; however, she declined and continued to be followed up closely by the oncology and pulmonary team. Our patient is unique due to the following features: *BRCA1* mutation associated with bilateral breast and ovarian cancer status post-resections and chemotherapy; PET-positive lesions in pulmonary parenchyma and mediastinal lymph nodes; elevated CA 15-3 and CA 27-29; and non-necrotizing granulomas on TBBX and EBUS-TBNA. Differential diagnoses included metastatic breast cancer and sarcoidosis.

## Discussion

Invasive ductal carcinoma associated with non-caseating epithelioid granulomas was first described by Oberman in 1987 [6]. In his series, the granulomas were restricted to the carcinoma, and no granulomatous response was evident in regional lymph nodes. None of the patients had clinical evidence of systemic granulomatous disease, although one patient was later found to have hepatic portal granulomas. Subsequently, case reports have revealed granulomatous stromal response in breast cancers [7-10]. These granulomas possibly reflect an immunological response to tumor antigens [11,12]. However, in the absence of clear evidence of malignant cells, other conditions resulting in granulomas must be excluded. Our patient had a history of treated tuberculosis. She had no constitutional signs of tuberculosis and pathology and cultures for acid-fast bacilli were negative. Fungal cultures were also negative. This led to the question of can this be sarcoidosis.

Sarcoidosis is a chronic inflammatory disease of unknown etiology, which can involve different organs and systems. Sarcoidosis can affect the breasts [13]. Breast sarcoidosis is extremely rare as an isolated extrapulmonary involvement [14]. Pulmonary sarcoidosis in patients with breast cancer is rare and can present at the same time causing a diagnostic dilemma leading to misdiagnosis and incorrect treatment [15,16]. Chen et al. reported a case series of patients with breast cancer and sarcoidosis. In their series of five cases, sarcoidosis preceded breast cancer in 50% of the cases, appeared after breast cancer in 25%, and occurred in tandem in 25%. In their literature review, 66 patients presented with both sarcoidosis and breast cancer. Sarcoidosis preceded breast cancer in 31 cases, followed it in 23 cases, and appeared concurrently in 10 cases [17]. In a recent review of 20 patients with sarcoidosis and breast cancer, in 12 cases breast cancer preceded sarcoidosis by 52 months, in four sarcoidosis preceded breast cancer by 200 months, and in another four both presented concurrently [18].

Differential diagnoses of elevated CA 15-3 and CA 27-29 include metastatic breast cancer and sarcoidosis-associated elevation. CA 15-3 and CA 27-29 are generally used to monitor chemotherapy in patients with stage IV breast cancer. In this setting, these biomarkers should not be used alone but in combination with imaging [19]. Other causes of elevation of these markers include both malignant and benign conditions. Malignancies other than breast resulting in elevated levels include lung, colon, pancreas, liver, ovary, cervix, and endometrial cancers. Benign conditions resulting in elevated levels include chronic hepatitis, liver cirrhosis, tuberculosis, systemic lupus erythematosus, and sarcoidosis. Pulmonary disorders associated with elevated CA 15-3 include interstitial lung disease, hypersensitivity pneumonitis, and SARS-CoV-2 pneumonia [20-22]. In a case report, Türk et al. reported a correlation between CA 15-3 levels and disease activity in sarcoidosis in a patient with a history of breast cancer [23].

PET is an imaging modality primarily used in the field of oncology. In patients with pulmonary nodules, an SUV of 2.5 is generally used as a cutoff value for diagnosing pulmonary malignancies [24]. Sarcoidosis, granulomatosis with polyangiitis, aspergillosis, and tuberculosis can mimic malignancy with an SUV >2.5. FDG PET has been well-studied in sarcoidosis. FDG PET/CT is neither a first-choice modality for the diagnosis of sarcoidosis nor a technique of choice for screening; however, it may be useful for assessing cardiac involvement and response to treatment [25]. In a retrospective study of 188 FDG PET scans performed for 137 patients with proven sarcoidosis, 139 scans had positive findings, with SUV ranging from 2.0 to 15.8 [26]. Various studies have shown that the degree of SUV change is a good objective tool in monitoring response to therapy [27-29]. Early metabolic response to systemic corticosteroid treatment may be used as a tool in the establishment of a final diagnosis when sarcoidosis is suspected in a cancer patient and can differentiate cancer from sarcoidosis in the case of coexisting diseases [30,31].

**Figure 3:**
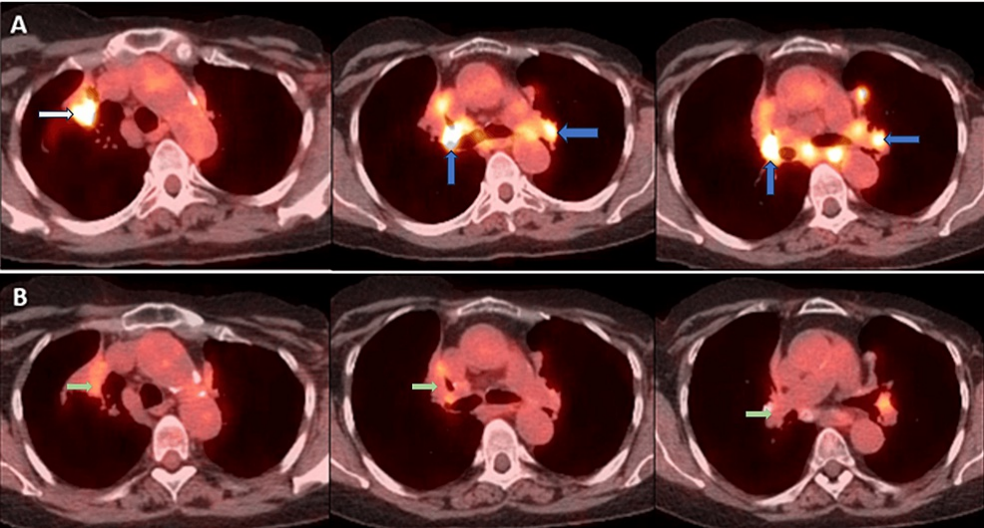
Representative sections of axial views of PET/CT scans showing a decrease in SUV. (A) PET/CT before bronchoscopy. (B) PET/CT seven months after starting oral prednisone therapy. White arrow showing lesion in the right upper lobe. Blue arrows showing PET-positive mediastinal lymphadenopathy. Green arrows showing reduced SUV uptake in the lesion and mediastinal lymphadenopathy after seven months of steroid treatment. PET/CT: positron emission tomography/computed tomography; SUV: standardized uptake value

A repeat CT three months after the initial visit did not reveal worsening of infiltrates or mediastinal lymphadenopathy. The patient continued to complain of shortness of breath. She was started on oral prednisone therapy for suspected sarcoidosis and tumor markers were trended. Tumor markers revealed a declining trend and repeated PET scans performed while on steroids showed decreasing PET activity and near-total resolution of PET activity in mediastinal lymph nodes (Tables 1, 2). Figure 3 shows a comparison of PET scans before and while on steroids. This established a diagnosis of sarcoidosis by exclusion and therapeutic response. Our patient is being followed in our pulmonary clinic and continues to do well.

**Table 1:** Trend of tumor markers before and after prednisone. CA: cancer antigen

	Before prednisone	Prednisone started	Four months after prednisone
CA 15-3 (normal: <25.0 U/mL)	30 U/mL	60.3 U/mL	28.9 U/mL
CA 27-29 (normal <38.0 U/mL)	42 U/mL	37 U/mL	36 U/mL

**Table 2:** Comparison of PET scans before and while on prednisone. PET: positron emission tomography; SUV: standardized uptake value

PET SUV	Initial PET	Two months of prednisone	Seven months of prednisone
Right hilum SUV	14.4	8-9	4.3
Left hilum SUV	12.9	8-9	None
Right lower paratracheal area SUV	8.2	8-9	None
Subcarinal space SUV	9.2	8-9	None
Aortic pulmonary window SUV	11.3	8-9	None
Right lower lobe atelectasis/consolidation SUV	7.7	6.3	3.9

## Conclusions

Sarcoidosis is a complex and enigmatic inflammatory disease that affects multiple organ systems, posing significant diagnostic and therapeutic challenges. Sarcoidosis and breast cancer can coexist in the same patient. Sarcoidosis can precede the development of breast cancer, follow it, or present concurrently. This can result in misdiagnosis, resulting in therapeutic mistakes. Biopsy of the lesions suggesting granulomas without any evidence of malignancy may be the only clue as serum markers for breast cancer, e.g., CA 15-3 and CA 27-29, can also be positive in sarcoidosis. FDG PET cannot reliably distinguish between malignancy and sarcoidosis. Early PET/CT response to treatment may be used as a tool in the establishment of a final diagnosis.
